# Behind the mask: a critical perspective on the ethical, moral, and legal implications of AI in ophthalmology

**DOI:** 10.1007/s00417-023-06245-4

**Published:** 2023-09-25

**Authors:** Daniele Veritti, Leopoldo Rubinato, Valentina Sarao, Axel De Nardin, Gian Luca Foresti, Paolo Lanzetta

**Affiliations:** 1 Department of Medicine – Ophthalmology, University of Udine, Udine, Italy; 2 Istituto Europeo di Microchirurgia Oculare – IEMO, Udine, Italy; 3 Department of Mathematics, Informatics and Physics, University of Udine, Udine, Italy

**Keywords:** Artificial intelligence, Bias, Black-box, Cybersecurity, Data ownership, Deep learning, Distributional shift, Ethics, Liability, Machine learning, Medical liability, Ophthalmology

## Abstract

**Purpose:**

This narrative review aims to provide an overview of the dangers, controversial aspects, and implications of artificial intelligence (AI) use in ophthalmology and other medical-related fields.

**Methods:**

We conducted a decade-long comprehensive search (January 2013–May 2023) of both academic and grey literature, focusing on the application of AI in ophthalmology and healthcare. This search included key web-based academic databases, non-traditional sources, and targeted searches of specific organizations and institutions. We reviewed and selected documents for relevance to AI, healthcare, ethics, and guidelines, aiming for a critical analysis of ethical, moral, and legal implications of AI in healthcare.

**Results:**

Six main issues were identified, analyzed, and discussed. These include bias and clinical safety, cybersecurity, health data and AI algorithm ownership, the “black-box” problem, medical liability, and the risk of widening inequality in healthcare.

**Conclusion:**

Solutions to address these issues include collecting high-quality data of the target population, incorporating stronger security measures, using explainable AI algorithms and ensemble methods, and making AI-based solutions accessible to everyone. With careful oversight and regulation, AI-based systems can be used to supplement physician decision-making and improve patient care and outcomes.

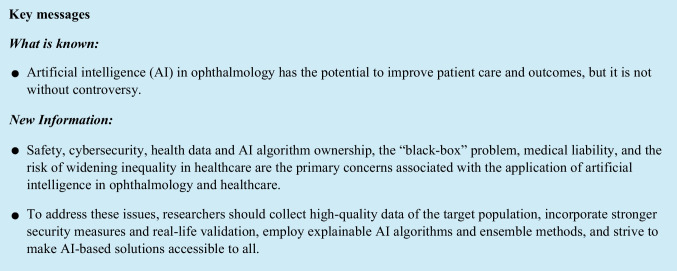

## Introduction

Artificial intelligence (AI) has the potential to revolutionize ophthalmology by making the diagnostic and decision-making process easier and faster through the analysis of large amounts of data [1]. AI methodologies have been applied to the analysis of ocular imaging, such as fundus photographs and optical coherence tomography, in the screening, diagnosis, and treatment of common ophthalmic diseases. While the use of AI in ophthalmology holds great promise, there are also a number of dangers and controversies that must be considered, including errors resulting from incorrect data synthesis, legal liability implications, cybersecurity and data ownership concerns, and the challenge of interpreting the output provided by AI software with “black-box” operation [2]. This narrative review aims to provide an overview of these issues and their implications for the widespread use of AI in ophthalmology.

## Methods

In this narrative review, we conducted a comprehensive search spanning January 2013 to May 2023 of both academic and grey literature to analyze the application of AI in ophthalmology and healthcare. Our search strategy was guided by a carefully selected set of keywords: “AI accountability,” “AI accessibility,” “AI in diagnostics,” “AI in medicine,” “AI in medical research,” “AI in patient monitoring,” “AI in prognosis,” “AI in treatment planning,” “AI transparency,” “artificial intelligence,” “bias in AI,” “clinical decision-making,” “cybersecurity,” “data privacy,” “deep learning,” “ethics,” “guidelines,” “healthcare,” “healthcare inequality,” “legal implications,” “machine learning,” “ophthalmology,” “patient care,” and “regulation.” Our search spanned five academic databases: ACM, PubMed, Nature-SCI, IEEE Xplore, and the AI Ethics Guidelines Global Inventory. Given the dynamic nature of the AI field, we also included grey literature, following the search methods outlined by Godin et al [3]. These additional sources included grey literature databases, Google Scholar, targeted website searches of known organizations and institutions, and the results from a prior environmental scan conducted by a team member (VS). Two investigators (DV and LR) selected documents based on their relevance to artificial intelligence, healthcare, ethics, and guidelines. Our focus was on the ethical, moral, and legal implications of AI in healthcare, aiming for a critical analysis rather than a comprehensive review. During the code mapping process, we used an abductive methodology, incorporating both inductive and deductive approaches, to identify and rename ethical issues based on the content of the selected documents. This approach allowed us to categorize the information into six primary issues associated with the use of AI in ophthalmology.

## Results

### Artificial intelligence, bias, and clinical safety

Recent advancements in AI have promoted the application of machine learning (ML) in clinical practice as a way to use data from past experiences to make inferences about new patients [1]. Although this approach showed some success, the use of ML systems has been shown to be prone to bias due to the phenomenon of “distributional shift,” where the ML system is inefficient at recognizing a relevant change in the actual context from the samples provided during its training [2]. This can lead to diagnostic errors due to unrepresentative training data, inadequate labeling of the patient’s outcome, inadequate definition of a gold standard diagnosis for the disease, and differences in the disease stage [4–6]. For example, the epic sepsis model (ESM) is an AI-powered sepsis prediction model, trained on large datasets, which demonstrated strong performance in its initial evaluation. However, the model performance dramatically fell when it was applied in the real-world. In an external validation study, this model only generated sepsis alerts for 843 cases (33%) on 2552 patients with clinically confirmed sepsis, missing 67% of the people with sepsis. Out of 6971 ESM sepsis alerts, only 843 (12%) were correct, resulting in 88% of false alarms [6].

The accuracy of ML model predictions can be strongly affected by bias in the training data. This bias can be caused by a variety of factors, including differences between the population of the training data and the target population (e.g., ethnicity, sex, age, comorbidities), variations in data quality (e.g., image quality on the fundus camera used for the screening of diabetic retinopathy (DR)), and differences in the way the data was collected [7, 8]. In ophthalmology, bias in ML training is a significant concern, especially when using ML algorithms for screening of DR among underprivileged populations. Incorrect training of ML models can lead to inaccurate results and potentially lead to inadequate care for patients in need [8]. For instance, the performance of automated DR algorithms varies considerably due to limited training data, heterogeneity in disease presentations, and suboptimal image quality [9, 10]. Moreover, ophthalmological ML-ready datasets are only available in a few countries, leaving a large number of countries unrepresented in training and validation cohorts [11]. Additionally, AI systems cannot capture the emotional components of diagnosis, such as the impact of a diagnosis on the patient’s quality of life. Esteva et al. compared ML systems to expert dermatologists on the ability to discriminate between benign and malignant skin lesions with the result that both humans and machines found it difficult to express a firm judgment, but because of the emotional aspect of a potentially life-threatening disease, human dermatologists were more prone to over-diagnose malignancy [12]. This aspect of human behavior is crucial for clinical safety and should be integrated into AI algorithms, such as in those used for high-risk DR screening. These algorithms should be trained not just with the end result, but also with the real-world impact of potential missed diagnoses.

### Cybersecurity

Data security is a major concern in healthcare, particularly given the current development of AI-powered solutions. Inadequate data security in healthcare can cause serious negative consequences, including potential data breaches, loss of patient information, potential misuse of sensitive information, and potential effects on patient’s health [13]. The security of digital medical devices, such as AI-based devices with diagnostic or therapeutic software, is becoming a critical issue in healthcare. The strongest motivation of healthcare systems hackers is the financial gain because the sale of health data is extremely valuable but should even be considered the political gain and the potential aim of controlling lives in a form of cyberwarfare [14]. In 2021, 45 million individuals in the US have been affected by healthcare attacks, a threefold increase from 2018 [15]. According to an annual report by “Comparitech”, ransomware attacks cost the healthcare industry $20.8 billion in 2020 in the USA alone [15]. An emblematic example of how the intrusion into AI algorithms in healthcare can have serious consequences for patients was reported by Zhou et al. [16]. These authors performed a study to investigate the behaviors of an AI breast cancer diagnosis model under adversarial mammogram images. They found that the adversarial mammogram samples fooled the AI model to output a wrong diagnosis in 69.1% cases that were initially correctly classified [16]. Furthermore, the Food and Drug Administration has reported that the “MiniMed™ 508” and the “MiniMed™ Paradigm” implantable insulin pumps, manufactured by the American company Medtronic, were vulnerable to external manipulations on the insulin release settings [17]. CyberMDX, an American cybersecurity company, also discovered previously undocumented vulnerabilities in the Alaris Gateway workstation pumps used in intravenous therapies, allowing hackers to alter the administration of life-saving treatments [18]. These examples highlight the urgent need to improve the security of computerized medical devices and AI software, to protect patients from potential risks. In the field of retinal diseases, for example, unauthorized access to AI systems developed to form a plan of optimal photocoagulation arrangement for retinal laser coagulation for each case could lead to manipulation of data or algorithms, potentially leading to incorrect diagnoses or treatment plans [19].

### Health data and AI algorithms ownership

In recent years, the healthcare industry has experienced the emergence of several corporate entrants, including digital technology companies, such as Google, Microsoft, IBM, and Apple. These companies have invested heavily in the development and use of AI technologies in healthcare, resulting in a number of innovative products and services. Google Health (Mountain View, California, USA) was launched in 2006, providing a repository of health records to connect doctors, hospitals, and pharmacies directly. Microsoft (Redmond, Washington, USA) followed in 2007 with “Microsoft-Healthvault,” a web-based personal health record to store health and fitness information. In 2019, Apple Inc. (Cupertino, California, USA) developed an algorithm for an apple watch application which was validated in the “Apple Heart Study” conducted at Stanford University [20]. Deepmind (a British artificial intelligence subsidiary of Alphabet Inc.) collaborated with the Moorfield Eye Hospital in 2020 to develop an AI system to predict whether a patient with unilateral neovascular AMD will develop neovascular AMD in the second eye from the analysis of OCT scans [21]. In July 2021, Google announced the “Healthcare Data Engine,” an end-to-end solution for healthcare and life sciences organizations to harmonize data from multiple sources for AI advanced analytics. However, these investments have raised some criticism. In July 2017, a deep-seated concern arose over the 2015 transfer of 1.6 million identifiable patient records from the London-based Royal Free NHS Trust to Google’s DeepMind health unit, without the patients’ explicit permission for their data to be shared [22]. This has opened a debate in the scientific community regarding the deliberate use of personal health data. The ownership of health data and responsibility for it are unresolved questions, with bioethics literature commonly asserting that patients should own their data and have the right to make decisions about its access [23–25]. The issue of health data and AI algorithms ownership is a critical concern in the field of ophthalmology. For instance, the development and application of AI algorithms, such as Eye2Gene, for diagnosing inherited retinal diseases (IRDs) have raised questions about who owns the data and the algorithm itself. Eye2Gene, an AI algorithm designed to accelerate the diagnosis of IRDs, utilizes patient retinal images to predict causative genes. The algorithm is trained on datasets from multiple hospitals and validated on external datasets. However, the ownership of the data used for training and validation, as well as the AI algorithm itself, is not explicitly stated. This situation underscores the need for clear policies and regulations regarding the ownership and use of health data and AI algorithms in ophthalmology [26]. Companies and institutions have started to consider patients as “data traders,” who provide information about their health in exchange for remuneration [27]. Online platforms, such as “The Savvy platform,” have been created to enable patients to sell and share their medical data. However, once the data has been shared, patients have no control over its secondary use.

### The “black-box” problem

AI systems used to interpret data and provide decisions based on that data have recently been subject to much debate regarding the “black-box problem” [28]. This concept refers to the opacity of the AI system, meaning that it is difficult to understand, predict, or systematically influence the way in which an input is transformed to an output. As a result, end-users are less likely to trust and cede control to machines whose workings they do not understand [29]. Some authors propose that the issue of transparency might be subordinate to the proficiency of AI in augmenting patient outcomes and overall public health. In this regard, Evans and colleagues for instance underscore that the capability of AI to substantially enhance the results of retinopathy of prematurity screening and treatment, particularly in resource-constrained environments, accentuates the potential advantages of AI in the healthcare sector despite the prevailing concerns related to transparency [30]. In contrast, other authors support white-box AI [31]. White-box AI is more transparent in its decision-making process, since it is based on simpler models such as linear regression and decision trees that are significantly easier to interpret. However, these models provide less predictive capacity and cannot always model the inherent complexity of the dataset. “Black-box” AI systems, on the other hand, are based on more complex algorithms and are more efficient and accurate than “white-box” models and are particularly useful in the field of image analysis and processing (as in the ophthalmology field) [32]. To increase trust, solutions such as estimating uncertainty of predictions and mimicking ophthalmologists’ clinical reasoning can be employed [33, 34]. Abràmoff et al. developed a two-stage ML system that first learns to detect lesions considered relevant by ophthalmologists (microaneurysms, exudates, etc.) and then it bases its predictions on these detections. Because these models mimic ophthalmologists’ clinical reasoning, they are more likely to be adopted in the clinical practice [35]. Class activation mapping is a technique that can be used to understand how and why a model makes a particular decision. This technique produces a heatmap of the input image which highlights the areas of the image that most strongly contribute to the model’s decision [36, 37]. While these techniques are instrumental for image-based models, there are also methods available to interpret the decision-making process for non-image data. Partial dependence plots (PDPs) and techniques like SHAP (SHapley Additive exPlanations) and LIME (Local Interpretable Model-Agnostic Explanations) provide robust ways of understanding how each feature influences predictions. PDPs show the effect of a feature on predictions, SHAP provides a consistent measure of feature importance, and LIME explains predictions by approximating the model locally with an interpretable model. In 2017, the European Union proposed the “Right for an Explanation” as an aim for AI applications utilizing personal data to make diagnostic or therapeutic decisions regarding individuals. This right is intended to ensure that patients and ophthalmologists comprehend the basis for such decisions, and to ensure the transparency and accountability of the AI system [38].

### Medical liability

Ophthalmologists have expressed concerns regarding the potential medical liability arising from errors in AI-based decision-making, particularly in the diagnosis and management of major eye conditions such as diabetic retinopathy, glaucoma, age-related macular degeneration, and cataracts. The fear of medical malpractice acts as a significant deterrent to the adoption of AI in clinical practice, despite its potential to improve diagnostic accuracy and efficiency [39].

The legal implications of AI-based medical decision-making are complex and multi-faceted. Who is to be held responsible in case of errors? The clinician, the software developer, the software rights holder, or the hospitals and institutions that adopted the system? Currently, AI-based software is not a component of the common medical standard of care. If a clinician follows an incorrect treatment recommendation given by AI-based software, and this leads to harm for the patient, the clinician is likely liable for medical malpractice. This is because AI-based software is usually considered a tool under the control of the healthcare professional, who must make the final decision. Therefore, clinicians could be held accountable even when they had faith in the black-box algorithms. In the USA, initiatives have been made to apply “product liability” laws to healthcare AI software, but various US courts have been reluctant to enforce these laws to healthcare AI algorithm developers due to the current perception of AI systems as confirmatory tools to support clinicians in their final decision [40, 41]. Others support the “negligent credentialing” theory as a potential avenue of liability for hospitals, should they fail to adequately review and verify the specifics and reliability of the AI systems they employ [42]. In 2017, the European Parliament released a resolution titled Civil Law Rules on Robotics. This document proposed the “human in command approach,” a principle ensuring that decisions pertaining to healthcare planning and treatment remain with humans and do not rely solely on AI tools. This principle was further developed in the European Commission’s White Paper on AI, released in 2020. This paper stated that the autonomous behavior of AI during their life cycle could cause significant changes to the software or algorithm, which could have a direct impact on safety and necessitate continuous risk assessment. Additionally, the Civil Law Rules on Robotics suggested that in cases of AI medical errors, responsibility should be proportional to the instructions given to the machine and its level of autonomy — the greater the learning capacity or decisional autonomy of a machine, the greater the responsibility of its human trainers should be [43].

### Lack of AI democratization and widening inequality in healthcare

In the context of healthcare, democratization refers to providing universal access to AI-based solutions and ensuring that everyone can benefit equally from this technology. The potential for disparate impact of AI-based solutions on underprivileged groups is a major concern. Research indicates that AI-based solutions can be biased in favor of the privileged and wealthy populations. This means that the AI-based solutions may not be equally accessible to those from other backgrounds. Furthermore, AI systems are only as good as the data used to train them. If the data used to train the AI system does not adequately represent the diversity of the population, then the AI system will produce biased outcomes [1, 44, 45]. For example, if the data used to train the AI system comes predominantly from Caucasian patients, then the AI system may be less accurate in detecting eye diseases in other ethnicities.

## Discussion

The use of AI in ophthalmology has been gaining increased attention in recent years due to its potential to improve patient care and outcomes. AI-based technologies have been found to provide accurate and timely diagnosis and management of eye diseases, as well as to reduce medical errors. However, the use of AI in ophthalmology is not without controversy. AI-based systems may not be able to account for the complexity of human decision-making, which can lead to errors or bias, leading to harmful consequences for vision and expose ophthalmologists to liability issues. This narrative review identified and evaluated the controversial aspects and implications of AI use in ophthalmology through the analysis of six key issues. Potential solutions to address them are:

1. Bias and distributional shift can be limited by ensuring that the population of the training data matches the population of the target population, by collecting data of high quality, and by using standardized data collection methods. Researchers should use methods such as stratified sampling and cross-validation with the aim to ensure that the results of ML models are accurate and reliable, upholding alignment with current clinical evidence. Real-life validation of a model’s performance is also needed to guarantee that the AI system is up to the task of making accurate and reliable diagnoses in a real-life setting. Additionally, the AI system must be tested in a variety of different settings to allow it to perform consistently in different environments [2, 44].
2. Healthcare organizations should adopt stronger security measures for their data infrastructure, such as encryption and authentication protocols, monitoring and logging systems, and AI-powered solutions certified by third-party organizations. Misuse of health data can lead to a breach of confidentiality and violation of patient privacy, so a secure approach to training AI systems is necessary. Federated learning is the ideal approach for ophthalmology and healthcare due to its capacity to protect patient data while still enabling the AI system to learn [4, 46]. Decentralizing the learning process ensures that data remains local and is not exposed to any third party. As such, model updates can be distributed to each device to improve performance while protecting patient data.
3. To ensure the ethical use of AI-based ophthalmology tools, there is a need for greater transparency in how patient data is collected and used. This should include clear guidelines on data handling and storage, as well as consent forms which explicitly outline how the data will be used. In addition, independent reviews of the algorithms used to power such applications should be conducted on a regular basis to ensure accuracy and fairness.
4. Strategies to address the black-box issue of AI in ophthalmology should be supported, in order to increase the confidence of the medical society in the clinical decisions suggested by the algorithms. One such strategy is to use explainable AI algorithms, which are designed to provide insight into the decision-making process of AI-based algorithms [5, 47]. Explainable AI algorithms are based on the idea that AI-based decisions should be explainable and interpretable, allowing medical professionals to better understand the decisions being made by the AI-based algorithms. Additionally, explainable AI algorithms can be used to provide better insight into the behavior of the algorithm and to identify potential bias or errors in the decision-making process. Another strategy is the use of ensemble methods. Ensemble methods involve combining multiple AI-based algorithms to create a more accurate and reliable decision-making process [6, 48].
5. We believe that AI-based healthcare solutions should be evaluated and approved by appropriate regulatory bodies, country-specific medical boards, and scientific societies as part of the “standard of care.” This would not only allow doctors to use the full potential of AI systems, but also provide a legal safety framework in the event of AI-related medical malpractice. However, this raises the additional question of whether manufacturers and sellers of AI-related products should be held liable under product liability law. Whatever the outcome, it is clear that the use of AI in ophthalmology requires careful consideration of legal and ethical implications.
6. Ensure that AI-based solutions are made accessible to everyone and that any potential for disparate impact on underprivileged groups is minimized. AI systems must be trained on large datasets that accurately represent the diversity of the population, which may be particularly challenging for rare diseases with limited data.

In conclusion, the use of AI in ophthalmology has the potential to improve patient care and outcomes, but it is not without controversy. It is important to consider the ethical and safety implications of AI-based decision-making, and to ensure that any decision made is in the best interest of the patient.

## Data Availability

Not applicable.
